# With thanks to our 2024 peer reviewers

**DOI:** 10.24095/hpcdp.45.3.03

**Published:** 2025-03

We are grateful to the following individuals for their significant contribution to*Health Promotion and Chronic Disease Prevention in Canada*as peer reviewers in 2024. Their expertise ensures the quality of our journal and promotes the sharing of new knowledge among peers in Canada and internationally.

Timothy Adair

Alimot Afolabi

Williams Agyemang-Duah

Md Sabbir Ahmed

Laura Anderson

Sharon Anderson

Suzanne Archie

Oluwagbohunmi Awosoga

Lynda G. Balneaves

Geoff Bardwell

Jennifer Baumbusch

Maulik Baxi

Kathy Belton

Ghose Bishwajit

Krysten W. Bold

Sue Booth

Vincent Gosselin Boucher

Avik Chaterjee 

Sitong Chen

Marie-Hlne Chomienne 

Jennifer Couturier

Samantha Cukier

K. Michael Cummings

Julie Darbyshire

Carolyn Dewa

Erica Di Ruggiero

Warren Dodd

Amanda Doggett

Shilpa Dogra

Isabelle Dor

Peter Driezen

Kayla Ellefsen

Tracy Everitt

Julie Faieta

Canisius Fantodji

Carol Fenton

Jonathon Fowles

Brianna Frangione

Sid Frankel

Genevive Garipy

Rochelle Garner

Vincent Gosselin Boucher

Shannon Gravely

Mikael Anne Greenwood-Hickman 

Emmanuel Guindon

Margareeta Hakkinen

Jillian Halladay

Barb Hamilton-Hinch

Miriam Harris

Jamie Hartmann-Boyce

Nancy Henderson

Katya Herman

Leah Hoffman

Melissa Holt 

Amy Hsu

Stephen Hunter

Jennifer Irwin

Leonard Jack, Jr.

Susan James

Euzebiusz Jamrozik

Feng Ji

Andrea Johnston

Akwatu Khenti

Erin Knight

Jordan Kuiper

Krim K. Lacey

Justin Lang

Caillin Langmann

Pamela Leece

Joan Lindsay

Julian Little

Lisa Lix

Yona Lunsky

Chris MacDonald 

Buuma Maisha

Bridget Maloney-Hall

Ian McConnachie

Mary McKenna

Peter Milgrom

Shawn Mondoux

Howard Morrison

Daniel T. Myran

Candace Nykiforuk

Dominika Ochnik

Jennifer O'Loughlin

Jeff Ondocsin

Neil Ortmann

Theone Paterson

Scott Patten

Linda Pederson

Paul Peters

M. Kathy Pichora-Fuller

Milo Puhan

Amity Quinn

Hudson Reddon

Justine Renard

Teodora Riglea

Karen Roberts

Yujiro Sano

Adam Sherk

Christopher Shields

Isabelle Simard

Jiraporn Sri-On

Clifford Stevenson

Jessica Stroope

Yves Terrat

Franois Thriault

Jason Thompson

Stephanie Toigo

Joslyn Trowbridge

Justin Tyndall

Chrystal Watson

Murray Weeks

Robert Wellman

Cliff Whetung

Bianca Wilhelm

Eryn Wright

